# The effect of natural sounds on the anxiety of patients undergoing coronary artery bypass graft surgery

**DOI:** 10.1186/s13741-017-0074-3

**Published:** 2017-11-15

**Authors:** Mohammad Javad Amiri, Tabandeh Sadeghi, Tayebeh Negahban Bonabi

**Affiliations:** 1 School of Nursing and Midwifery, Rafsanjan University of Medical Sciences, Rafsanjan, Iran; 2 Non-Communicable Diseases Research Center, Rafsanjan University of Medical Sciences, Rafsanjan, Iran; 3 Department of Pediatric Nursing, School of Nursing and Midwifery, Rafsanjan University of Medical Sciences, Jomhori St., Rafsanjan, 7718796755 Iran; 4 Social Determinants of Health Research Center, Rafsanjan University of Medical Sciences, Rafsanjan, Iran; 5 Deparment of Community Health Nursing, School of Nursing and Midwifery, Rafsanjan University of Medical Sciences, Rafsanjan, Iran

**Keywords:** CABG, Music therapy, Natural sounds, Anxiety

## Abstract

**Background:**

This study aims to investigate the effect of natural sounds on the anxiety of patients undergoing coronary artery bypass graft surgery (CABG).

**Methods:**

In this clinical trial, 90 patients, who were candidates for CABG in an urban area of Iran, were selected and randomly assigned to intervention and control groups by the minimization method. In the intervention group, natural sounds were broadcast through headphones for 30 min. In the control group, headphones connected to a silent device were used. The research instruments were a demographic questionnaire and the Spielberger State-Trait Anxiety Inventory (STAI). These were used before the intervention, 30 min after the music, and before the surgery in the waiting room for both groups. Data was analyzed using SPSS software.

**Results:**

The mean anxiety level of the intervention group has been found to be significantly lower than that of the control group half an hour after the intervention as well as in the waiting room in the preoperative period (*p* = 0.001). Moreover, the mean anxiety of the intervention group decreases, while it increases for the control group over time (*p* < 0.001).

**Conclusion:**

Natural sounds can be used as a non-pharmacological way to reduce the anxiety of patients undergoing CABG.

**Trial registration:**

IRCT2017011723190N3, Registered 1 March 2017.

## Background

Coronary artery bypass graft surgery (CABG) is one of the most valuable and effective methods for treating coronary artery diseases, but it is also one of the most stressful events that can occur during a person’s lifespan (Smeltzer et al. 2012). Patients undergoing CABG may suffer from psychological problems such as anxiety, depression, worry, and fear. These problems start as early as CABG is selected as a therapeutic approach and continue until the moment of discharge (Dehghani et al. 2013).

Symptoms such as fatigue, muscle weakness, palpitations, chest pain, headache, and sweating are negative effects of anxiety (Kipnis et al. 2016). Evidence suggests that candidates for surgery often worry about the procedure and fear the outcome (Thompson et al. 2014). Possible surgical pain, loss of control during surgery, changes in the body image, unpleasant diagnosis, worry about lack of rehabilitation, and worry for family members and occupation are among the preoperative anxiety-related factors (Kipnis et al. 2016).

With its risks, complications, and prognosis, cardiac surgery causes anxiety on a wider scale and sometimes causes patients to be defeated even before the surgery (Shojaee et al. 2014). Preoperative procedures, worry about the outcome, worry about lack of control over the situation, the risk of death, unfamiliar and unpleasant situations, and the expectation of undesirable results can contribute to anxiety in patients undergoing CABG (Padamanabhan et al. 2005). These issues cause postoperative pain, increase the need for analgesia, prolong hospital stay, delay discharge, have an overall effect on the induction and recurrence of anesthesia, and reduce the postoperative satisfaction in patients (Mirbagher et al. 2011). According to Krannich et al. (2007), patients undergoing CABG experience some degree of anxiety before and after surgery. These patients are very anxious due to the relationship between heart and life that exacerbates their excitement (Navarro et al. 2011).

Anxiety increases the heart rate, cardiac output, and the need for oxygen. It is necessary to reduce anxiety (Dehghani et al. 2013). One of the methods used in this field is music therapy (Sahmoddini et al. 2014), which is a complementary treatment that improves the patient’s well-being by improving the threshold of stress and eliminating negative emotions, adjusting internal processes, and making them feel relaxed. Music with a slow, uniform, and repetitive rhythm may induce sleep or relaxation in the listener (Chang et al. 2008). Creating a therapeutic environment helps the patient to move from anxious and negative thoughts toward things that are pleasant and desirable (Fredriksson et al. 2009). In recent studies, music therapy has been found to reduce patient anxiety (Kipnis et al. 2016; Thompson et al. 2014; Lee et al. 2011; Motahedian et al. 2014; Maeyama et al. 2009; Han et al. 2010; Wakim et al. 2010). However, some other studies have reported no meaningful results for music therapy in terms of the level of anxiety and vital signs of patients (Dileo 2006; Ebneshahidi and Mohseni 2008; Bradt et al. 2013; Sendelbach et al. 2006; Nilsson et al. 2009).

One of the varieties of music is nature sounds. The use of natural sounds, such as the chirping of birds or the sound of rain and water, was introduced for the first time in 1984 to treat anxiety and pain in patients (Aghai et al. 2014). There is a vast body of research on the effects of music on the anxiety, pain, and vital signs of patients, but limited studies have been conducted on the effects of nature sounds. In Iran, no study has been conducted on this particular subject except the one by Aghaie et al. (2014), which investigated the effect of natural sounds on anxiety in CABG patients during the weaning of mechanical ventilation. Therefore, by considering the importance of the issue of anxiety in patients undergoing CABG and the contradictory results of studies on the effect of music, this study aims to investigate the effect of natural sounds on the anxiety of patients undergoing CABG.

## Methods

This study was a clinical trial that was registered at the Iranian Registry for Clinical Trials with the code IRCT2017011723190N3. The research population included patients undergoing CABG hospitalization in an intensive care unit in an urban area of Iran. We calculated the study sample size by comparing two mean formulae with an effect size of 7 for anxiety and a standard deviation of 10 based on previous studies (McClurkin and Smith 2016). The level of significance was set at *p* < 0.05, while the study power was assumed to be 90%. We determined a sample size: *n* = 43.82. Considering the possibility of sample loss, 45 patients were allocated to each group. Alertness and being aware of the time and place, the age of over 18, undertaking heart surgery for the first time, having no pain, and having no evidence of organic fever during collection of information, taking no sedative, having no hearing problem, having no problem with the use of headphones, ability to participate in research and collaboration, signing the informed consent form, passing at least 2 h after admission, taking no beta blockers, and taking no narcotic drug in the morning before the surgery with any route of consumption such as oral, sublingual, injection, and inhalation were the inclusion criteria for this research study. The unwillingness to participate in research, interrupting music while performing the experiment, and the need to use sedative and narcotic drugs during voice playback or before completion of the second turn of the questionnaire formed the exclusion criteria.

As many as 90 candidates for CABG were selected based on inclusion and exclusion criteria by purposeful sampling. They were assigned to two intervention and control groups using the method of categorization through the minimization method (Pandis et al. 2011). In this method, the patients were initially categorized based on key variables such as anxiety score and gender. Afterward, the first participant was chosen from among those patients who met the inclusion criteria and then placed in the intervention or control group by the toss of a coin, while other participants were allocated to the study group with a lower number of variables (anxiety score and gender). In case of equality, random allocation was repeated.

The data collection tools comprise a demographic questionnaire for patients (age, sex, education, marital status, duration of disease) and the Spielberger State-Trait Anxiety Inventory (STAI). The reliability of the demographic questionnaire was determined by the content validity method as well as the opinion of professors and experts. The STAI is a commonly used measure of trait and state anxiety. Form Y, its most popular version, has 20 items for assessing trait anxiety and 20 for state anxiety. Internal consistency coefficients for the scale have ranged from 0.86 to 0.95, while test-retest reliability coefficients have ranged from 0.65 to 0.75 over a 2-month interval (Spielberger et al. 1983). In this study, we investigated state anxiety that relates to the apparent anxiety (expressing the patient’s feelings at the current moment and at the time of filling in the questionnaire) by using 20 questions. Scoring this scale includes scores from 1 to 4. The scores are reversed for reverse questions (i.e., questions 1, 2, 5, 8, 10, 11, 15, 16, 19, 20) so that it includes from never (4) to many times (1). The range of the score remains between 20 and 80. In Mahram’s study, the reliability of the research tool for the normal group (600 people) was 0.90 according to Cronbach’s alpha, while it was estimated in the standard group (130 persons) as 0.94 (Mahram 1995).

The method was implemented in such a way that the researcher (first author) was referred to the hospital according to the cardiac surgery program in the night shift before the operation and following the selection of samples according to the inclusion and exclusion criteria. Consent was obtained from the participants, and the demographic data form and the Spielberger STAI were completed to assess the level of state anxiety through face-to-face interviews. The patients were then randomly assigned to the intervention and control groups. For the intervention group, nature sounds were played for 30 min via headphones in order to remove additional sounds. In the control group, headphones were connected to a silent device and were placed on the ears of the patients. Thirty minutes after the completion of the music playing and on the morning of the operation day in the waiting room, the anxiety questionnaires were completed by the members of the two groups. Nature sounds used in this study were the artwork “Sounds of Nature” recorded by Dr. Arnd Stein in 2011. The sounds included birds’ chirping, roar of the sea, river and jungle sounds, and the sound of rain. The sound was broadcast by a 25–30-dB Sony MP3 player (as advised by the hearing aid) for 30 min for the intervention group. It should be noted that for preventing any transmission of infection from the headphones, they were disinfected after each use and disposable covers were used for them.

Finally, the data was analyzed using descriptive statistics, such as mean and standard deviation, and analytical statistics including the independent *t*-test and repeated measures ANOVA with SPSS software version 18. The significance level was less than 0.05.

## Results

The demographic results indicate that in the intervention group, the mean age of patients was 58.61 ± 9.55 years and the mean duration of the disease was 9.51 ± 5.02 years. In the control group, the mean age of patients was 57.71 ± 9.88 years and the mean duration of their occurrence was 11.66 ± 3.99 years; they were homogeneous in terms of demographic characteristics (Table 1).

**Table 1:** Comparison of demographic characteristics of patients in intervention and control groups

Demographic data		Intervention group number (%)	Control group number (%)	*p* value
Age (years)	>40	1(2.3)	4(8.9)	0.28^a^
	40–60	24(53.3)	14(31.1)	
	<60	20(44.4)	27(60)	
Gender	Man	27(60)	29(64.4)	0.66^b^
	Woman	18(40)	16(35.6)	
Marital status	Married	39(86.7)	34(95.6)	0.13^b^
	Single	6(13.3)	2(4.4)	
Disease duration (years)	>5	12(26.7)	9(20)	0.91^a^
	5–15	28(62.2)	34(75.6)	
	<15	5(11.1)	2(4.4)	

^a^Mann-Whitney *U* test

^b^Chi-square

Based on the results, no significant difference was found between the two groups before the intervention (independent *t*-test, *p* = 0.44). However, there was a significant difference between the two groups after the intervention and the mean anxiety level in the intervention group was lower (independent *t*, *p* < 0.05). An intra-group analysis of variance with repeated measures was used: this resulted in no significant effect on the time (*p* = 0.46). The interactive effects of time and group were also evaluated using the repeated measures analysis of variance. While the result was significant and showed a decrease in the mean anxiety of the intervention group, the mean anxiety of the control group increased (*p* < 0.001) (Table 2). Normal distribution of data was confirmed by using the Kolmogorov–Smirnov statistical test (*p* > 0.05).

**Table 2:** Comparison of mean anxiety level in patients of both groups before and after the intervention

Groups	Time of intervention			Results of repeated measures ANOVA	
	Before intervention	30 min after the intervention	In the waiting room	*p* value^b^	*p* value^c^
	Mean ± SD	Mean ± SD	Mean ± SD		
Intervention group	4.37 ± 47.66	7.21 ± 43.68	7.11 ± 42.95	0.34	0.001
Control group	5.17 ± 48.44	6.69 ± 50.57	6.78 ± 51.28		
*p* value^a^	0.44	0.001	0.001		

^a^Independent *t*-test (inter-group comparison)

^b^Repeated measures ANOVA (intra-group comparison)

^c^Repeated measures ANOVA (interactive effect)

## Discussion

Based on the results of this study, there was a significant statistical difference between the two groups in terms of playing natural sounds half an hour after and before surgery in the waiting room: the mean of anxiety in the intervention group was also lower. In the field of natural sounds, the results of the study by Aghaie et al. (2014) were consistent with the results of the present study—this showed that playing natural sounds can reduce anxiety and restlessness in CABG patients during the weaning of mechanical ventilation. Although this study investigated the effect of natural sounds in CABG patients, the difference between this and the present study lies in the domain of time: this study was conducted before the surgery and the study by Aghaie et al. was conducted postoperation.

Although a similar study was not found in terms of the use of natural sounds before CABG, music intervention has been studied in several studies and various results have been reported. For example, the results of McClurkin et al. (2016) are consistent with the results of the present study which showed that listening to music for at least 15 min reduced the anxiety of patients undergoing surgery. But the difference with the present study was in the research procedure: in addition to the control group, there was a group hearing music for 15 min and one group for 30 min: all patients were candidates for various surgical procedures (orthopedics, general, neurological, plastic, urological), and based on their interest they selected classical, religious, or nature music. However, in the present study, the effect of natural sounds music was studied and patients were candidates for CABG. The results of the study by Thompson et al. (2014) and Maeyama et al. (2009) also showed that playing music before the surgery could reduce the anxiety of patients; these are consistent with the findings of the present study, although the type of music and patients of the aforementioned studies are different from the present study.

Despite the results of this study, the findings from Ebneshahidi and Mohseni (2008), Sendelbach et al. (2006), and Nilsson et al. (2009) have no reported significant results from music intervention on the level of anxiety and vital signs of patients under surgery. Differences in the research population, type of music, patients’ condition, and music playing time can be mentioned as the reasons for the differences between the present study and the aforementioned studies.

Based on the results of the present study, the mean anxiety of the intervention group decreased but the mean anxiety of the control group increased; in the preoperative waiting room, the intervention group had lesser anxiety than the control group. In this regard, the results of Kipnis et al. (2016) are consistent with the results of the present study, which shows that playing music in the waiting room can reduce the level of anxiety in patients. Considering that music intervention on the night before the surgery can be more convenient and has less interferences with preoperative care: based on the results of this study, playing music on the night before surgery can be considered as an intervention to reduce preoperative anxiety. This is the strength of the present study over all the aforementioned studies.

Music can facilitate anxiety reduction due to its physiological and neurological effects. After studying 23 studies on ischemic heart disease, Bardt and Dileo (2013) stated that music has beneficial effects on blood pressure, heart rate, respiration, anxiety, and pain in people with this disease. Han et al. (2010) also found that relaxing music can reduce patient anxiety: their results are consistent with the results of this study. In Iran, due to cultural and religious beliefs, any kind of music is not pleasant to people; therefore, the music of nature’s sound, which is reminiscent of the natural habitats in the environment, is not in conflict with religious and cultural beliefs. Even in religious books such as the *Quran* and cultural books like *Divan Hafez*, thinking of nature and the consequences of thinking about it have been emphasized. These can help reduce this challenge.

Since the special environment of intensive care units increases the anxiety of patients due to factors such as sensory deprivation, severe medical noise, sleep deprivation, fear of diagnostic procedures, and separation from family members and friends (Rezaie et al. 2016), the results of this research can be used as a strategy by nurses to reduce the anxiety of patients undergoing CABG in intensive care units.

Physiological indices such as heart rates as well as blood pressure of these patients and the cortisol level taken at the time of the start of the surgery could have provided further evidence of the value of the administration of music to cardiac patients. These were not assessed in this study and are lost opportunities. Therefore, it is recommended that future studies be conducted as to assess these variables. This study was limited to patients with CABG. Lack of similar research on natural sound music limited the possibility of comparing the results of this study. So, doing similar studies in people with a different age range and other illnesses, comparing this kind of music with other types of music, and comparing the duration of the music playback are recommended for future studies.

## Conclusion

Natural sounds can be used as a way to reduce the anxiety of patients before surgery. Nurses can use this non-pharmacological approach along with other types of care to reduce the anxiety of patients before CABG.

## Abbreviations

CABG: Coronary artery bypass graft surgery
STAI: State-Trait Anxiety Inventory
